# Nanocolloidal albumin-IRDye 800CW: a near-infrared fluorescent tracer with optimal retention in the sentinel lymph node

**DOI:** 10.1007/s00259-012-2080-5

**Published:** 2012-02-17

**Authors:** Derrek A. Heuveling, Gerard W. M. Visser, Mattijs de Groot, Johannes F. de Boer, Marian Baclayon, Wouter H. Roos, Gijs J. L. Wuite, C. René Leemans, Remco de Bree, Guus A. M. S. van Dongen

**Affiliations:** 1 Department of Otolaryngology/Head and Neck Surgery, VU University Medical Center, De Boelelaan 1117, P.O. Box 7057, 1007 MB Amsterdam, The Netherlands; 2 Department of Nuclear Medicine & PET Research, VU University Medical Center, Amsterdam, The Netherlands; 3 Department of Physics and Astronomy, VU University, Amsterdam, The Netherlands

**Keywords:** Nanocolloidal albumin, IRDye 800CW, ICG, Sentinel lymph node, Near-infrared fluorescence, Quantum yield

## Abstract

**Purpose:**

At present, the only approved fluorescent tracer for clinical near-infrared fluorescence-guided sentinel node (SN) detection is indocyanine green (ICG), but the use of this tracer is limited due to its poor retention in the SN resulting in the detection of higher tier nodes. We describe the development and characterization of a next-generation fluorescent tracer, nanocolloidal albumin-IRDye 800CW that has optimal properties for clinical SN detection

**Methods:**

The fluorescent dye IRDye 800CW was covalently coupled to colloidal human serum albumin (HSA) particles present in the labelling kit Nanocoll in a manner compliant with current Good Manufacturing Practice. Characterization of nanocolloidal albumin-IRDye 800CW included determination of conjugation efficiency, purity, stability and particle size. Quantum yield was determined in serum and compared to that of ICG. For in vivo evaluation a lymphogenic metastatic tumour model in rabbits was used. Fluorescence imaging was performed directly after peritumoral injection of nanocolloidal albumin-IRDye 800CW or the reference ICG/HSA (i.e. ICG mixed with HSA), and was repeated after 24 h, after which fluorescent lymph nodes were excised.

**Results:**

Conjugation of IRDye 800CW to nanocolloidal albumin was always about 50% efficient and resulted in a stable and pure product without affecting the particle size of the nanocolloidal albumin. The quantum yield of nanocolloidal albumin-IRDye 800CW was similar to that of ICG. In vivo evaluation revealed noninvasive detection of the SN within 5 min of injection of either nanocolloidal albumin-IRDye 800CW or ICG/HSA. No decrease in the fluorescence signal from SN was observed 24 h after injection of the nanocolloidal albumin-IRDye 800CW, while a strong decrease or complete disappearance of the fluorescence signal was seen 24 h after injection of ICG/HSA. Fluorescence-guided SN biopsy was very easy.

**Conclusion:**

Nanocolloidal albumin-IRDye 800CW is a promising fluorescent tracer with optimal kinetic features for SN detection.

**Electronic supplementary material:**

The online version of this article (doi:10.1007/s00259-012-2080-5) contains supplementary material, which is available to authorized users.

## Introduction

The sentinel node (SN) procedure is a diagnostic staging procedure, which is applied in a variety of tumour types, including head and neck squamous cell carcinoma (HNSCC) [1, 2]. The identification and rough localization of the SN is generally based on the results of preoperative lymphoscintigraphy using planar or SPECT imaging, performed after peritumoral injections of a ^99m^Tc-labelled colloid (in Europe generally ^99m^Tc-Nanocoll).

To pinpoint the exact localization of the SN, so that the duration and extent of surgical exploration is minimized, intraoperative gamma probe-guided detection and blue dye lymphography are used. The gamma probe is used to guide the surgeon to the SN, which still contains sufficient amounts of radiocolloid at the time of surgery. The limitation of the gamma probe, however, is the lack of real-time visualization of the SN and information about SN depth. Moreover, as a result of high radioactivity arising from the injection site, detection of a SN close to the tumour may be difficult. Blue dye is injected in the same way as the radiocolloid, just before surgery, allowing real-time lymphatic mapping. Blue dye follows lymphatic vessels and accumulates in the draining lymph nodes staining them blue [3]. However, real-time detection of this blue staining is only possible if there is no overlying tissue. Moreover, blue dye is a relatively low molecular weight compound with a very poor retention in the SN and is therefore present for a short time. As a consequence, the use of blue dye is of limited added value in the head and neck area [4].

The use of near-infrared (NIR) fluorescence imaging might be an option for improving current clinical procedures. NIR fluorescence allows high-resolution dynamic imaging of superficial tissue layers without a radiation burden to the patient, and can be used for real-time intraoperative visualization of the location of the SN. In addition, in contrast to the blue dye-guided method, NIR fluorescence detection might be possible even if the SN is covered by tissue. Consequently, it might make the intraoperative use of the gamma probe and blue dye superfluous, especially when retention of the fluorescent tracer in the SN is good. At present, the only FDA-approved NIR fluorescent compound that has been extensively evaluated for SN detection is indocyanine green (ICG) [5–10]. ICG-guided SN detection has been proven to be feasible in, for example, breast cancer and skin cancer, with comparable or slightly better detection rates than conventional techniques [8–13]. In these studies, relatively rapid passage of ICG through the SN was observed, resulting in the detection of higher tier nodes when the identification time increased from, for example, 10 to 20 min [11].

IRDye 800CW is a promising next-generation NIR fluorophore. This NIR fluorophore shows less nonspecific binding and can, in contrast to ICG, be covalently conjugated to a broad range of biomolecules [14, 15]. This property allows conjugation of IRDye 800CW to colloidal human serum albumin (HSA) particles such those present in Nanocoll labelling kits (here referred to as nanocolloidal albumin), which is considered to be the most attractive carrier compound for SN detection (“gold standard”), since this ^99m^Tc-labelled colloid has been widely used for many years in the clinical SN procedures in Europe. The principles of IRDye 800CW conjugation to nanocolloidal albumin were described by Ohnishi et al. in 2005 [16] as part of a comparative preclinical evaluation. Since then, the SN concept has been established for solid tumours other than breast tumours and melanoma. Furthermore, IRDye 800CW is produced in a manner compliant with current Good Manufacturing Practice (cGMP). Clinical evaluation of IRDye 800CW is now possible, since toxicity studies did not reveal pathological evidence of toxicity [17] and, together with the filing of a Drug Master File at the US FDA, an Active Substance Master File has been filed with the Dutch regulatory authorities in support of an Investigational Medical Product Dossier for an IRDye 800CW-labelled targeted agent. Data from these studies will enable the filing of investigational new drug applications, e.g. nanocolloidal albumin-IRDye 800CW, in order to be able to start clinical exploration of the dye.

In the present study, a technical protocol was designed to produce covalently conjugated nanocolloidal albumin-IRDye 800CW in a cGMP-compliant way. Conjugation efficiency, purity, stability, fluorescence quantum yield and particle size of the nanocolloidal albumin-IRDye 800CW were determined. Proof-of-principle studies with this conjugate were performed in a rabbit lymphogenic metastatic tumour model, which mimics HNSCC [18, 19]. In these in vivo studies, ICG mixed with HSA was evaluated as a reference, since this combination is often used in clinical fluorescence imaging studies on SN detection.

## Materials and methods

### Materials

All reagents were purchased from Sigma-Aldrich (St. Louis, MO) unless otherwise stated. ICG (molecular weight 775 Da) was purchased from PULSION Medical Systems (München, Germany), and diluted to a concentration of 2.5 μg/μl by adding 0.9% NaCl. IRDye 800CW *N*-hydroxysuccinimide (NHS) ester (molecular weight 1,166 Da; LI-COR Biosciences, Lincoln, NE) was supplied by Westburg (Leusden, The Netherlands), and dissolved in DMSO to a concentration of 0.58 μg/μl. Nanocoll was purchased from GE Healthcare (Eindhoven, The Netherlands) as a kit for labelling with ^99m^Tc, containing amongst other components 0.5 mg lyophilized nanocolloidal albumin. The buffer used for nanocolloidal albumin-IRDye 800CW production consisted of 7.35 mg/ml sodium citrate with 0.7 mg/ml Tween-80 diluted in deionized (18 MΩ·cm) water, and is referred to here as Tween-citrate buffer (TCB). VX2 tumour cells were kindly provided by Dr. RJ van Es (Department of Oral and Maxillofacial Surgery, University Medical Center Utrecht, Utrecht, The Netherlands). Female rabbits (HsdIf:NZW), weighing 1.5–2.5 kg, were purchased from Harlan Laboratories (Hillcrest, Loughborough, UK).

### Preparation of nanocolloidal albumin-IRDye 800CW

IRDye 800CW was coupled covalently to nanocolloidal albumin according to the following procedure. TCB (1 ml) adjusted with 0.1 M Na_2_CO_3_ to pH 8.5, was added to a Nanocoll kit vial, and 0.5 mg of nanocolloidal albumin was purified by separating it from stannous chloride and other (small molecular) ingredients present in the kit (i.e. glucose, poloxamer 238, sodium phosphate and sodium phytate) by size-exclusion chromatography using a PD10 column (GE Healthcare Life Sciences) with TCB (pH 8.5) as eluent. The void volume and the first 1.5 ml were discarded; the next 2 ml contained the purified nanocolloidal albumin. Subsequently, to 1 ml eluate containing the purified nanocolloidal albumin (0.25 mg, corresponding to 3.7 nmol HSA building blocks, pH 8.5) was added 11.6 μg of IRDye 800CW-NHS ester (10 nmol in 20 μl DMSO; a 2.7 molar excess over HSA units that appeared to provide a product with optimal fluorescence yield), and the mixture was incubated for 2 h at 35°C. After 2 h, nonconjugated IRDye 800CW was removed by size exclusion chromatography using a PD10 column and TCB (pH 6.5) as eluent. The void volume and the first 1.5 ml were discarded; the next 2 ml containing the nanocolloidal albumin-IRDye 800CW conjugate was used for further experiments.

### Determination of conjugation efficiency

**Fig. 1:**
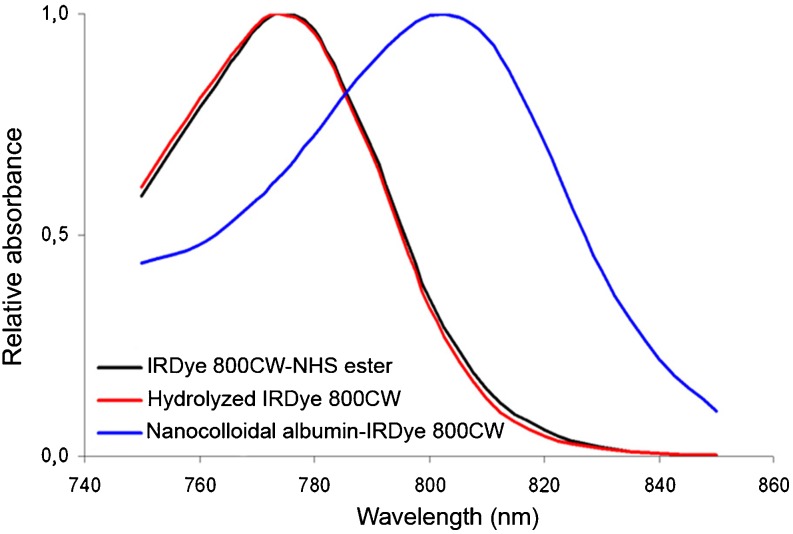
Absorbance spectrum of IRDye 800CW-NHS ester, hydrolysed IRDye800CW, and nanocolloidal albumin-IRDye 800CW

The efficiency of conjugation of IRDye 800CW to nanocolloidal albumin was determined based on absorbance measurements using an Ultrospec III spectrophotometer (Pharmacia, Biotech, Roosendaal, The Netherlands) at a wavelength of 774 nm. Since the absorption spectrum of IRDye 800CW changes upon conjugation to nanocolloidal albumin, calculations were based on the absorbance of free/hydrolysed IRDye 800CW in the solution. There were no differences in absorbance between the IRDye 800CW-NHS ester and the hydrolysed IRDye 800CW (Fig. 1). Due to their colloidal nature, direct HPLC analysis of nanocolloidal albumin particles was considered not to be an option.

Absorbance measurements were performed directly after addition of IRDye 800CW-NHS ester to the nanocolloidal albumin solution (Abs_start_) and after 2 h conjugation time. At the latter time, both nanocolloidal albumin-IRDye 800CW as well as free (hydrolysed) IRDye 800CW are present in the reaction mixture. In order to differentiate between conjugated nanocolloidal albumin-IRDye 800CW and free IRDye 800CW, a twofold volume excess of acetonitrile (MeCN) was added to the reaction mixture for precipitation of the nanocolloidal albumin. Subsequently, the absorbance of the supernatant (Abs_sup_) was measured, which had to be multiplied by 3 for correction of the dilution by MeCN. Conjugation efficiency was calculated using the expression:

$$ conjugation\;efficiency = \left( {1.0 - \frac{{3 \times Ab{s_{{\sup }}}}}{{Ab{s_{{start}}}}}} \right) \times 100\% $$

To prove that binding of IRDye 800CW to nanocolloidal albumin is based on covalent bonds and not on association, control measurements were carried out using hydrolysed IRDye 800CW. To this end, 20 nmol IRDye 800CW-NHS ester in 40 μl DMSO was added to 2 ml TCB (pH 8.5) and incubated for 2 h at 35°C, which resulted in >95% hydrolysis of the NHS ester (supplementary Fig. S1). Subsequently, the hydrolysed IRDye 800CW was added to a kit vial containing 0.5 mg nanocolloidal albumin and the mixture was incubated for an additional 2 h at 35°C. After measurement of absorbance, the nanocolloidal albumin was precipitated and the absorbance of the supernatant determined as described above. In addition, this method was also applied to determine the purity of the final nanocolloidal albumin-IRDye 800CW product and its stability as a function of time (measured over 120 h).

For further verification of the validity of the precipitation method, a reaction was performed using similar amounts of protein and IRDye 800CW as described above, but with native HSA instead of nanocolloidal albumin. With this approach HPLC analysis is also possible. After the 2-h reaction period, HPLC of the conjugation mixture was performed using a Jasco HPLC system equipped with a Superdex 200 10/30 GL size-exclusion column (GE Healthcare Life Sciences) with a mixture of 0.05 M sodium phosphate (pH 6.8), 0.15 M sodium chloride and 0.01 M NaN_3_ as the eluent at a flow rate of 0.5 ml/min. The area under the curve in the 780 nm channel of the hydrolysed IRDye 800CW peak at *R*_t_ = 50.4 min upon incubation in the presence of HSA was determined and compared with the area under the curve of hydrolysed IRDye 800CW incubated in the absence of HSA. The precipitation method as described above was then applied.

### Particle size measurements of nanocolloidal albumin-IRDye 800CW

For determination of the particle size, nanocolloidal albumin-IRDye 800CW was analysed by atomic force microscopy (AFM), which is a single-particle technique that has been used successfully to characterize a wide variety of nanometre-sized proteinaceous assemblies [20–22]. The atomic force microscope (Nanotec Electronica, Tres Cantos, Spain) was operated in jumping mode and all experiments were performed in liquid. Rectangular cantilevers (Olympus RC800PSA) with a nominal tip radius of 20 nm and spring constant of 0.05 N/m were used to image the colloidal particles. Colloidal samples were deposited on freshly cleaved mica substrate, and incubated for 10 min before analysis by AFM. As a reference, the native contents of a Nanocoll kit, diluted in TCB pH 6.5) to a concentration of 0.10 mg/ml nanocolloidal albumin were analysed. The height of immobilized particles was determined automatically using an in-house program written in LabView (National Instruments) to obtain the particle size distribution.

### Fluorescence properties

The fluorescence properties of nanocolloidal albumin-IRDye 800CW were determined using a FluoroMax-3 spectrofluorometer (Horiba Jobin Yvon, Edison, NY) and absorbance (corrected for background measurements) was measured using an UltroSpec 1100 Pro spectrophotometer (Amersham Pharmacia). Absorbance was always <0.1, in order to avoid confounding effects such as self-quenching and self-absorption. Measurements were performed in human serum, which was diluted fourfold with TCB (pH 6.5) in order to mimic the in vivo protein concentration of the lymphatic fluid, part of the interstitial body fluid, as well as in TCB (pH 6.5) alone. Comparative measurements were done for ICG. All measurements were carried out using freshly prepared IRDye 800CW-NHS ester (before conjugation) and ICG in order to minimize the possibility of degradation effects of ICG and IRDye 800CW. In addition, all spectrofluorometer measurements were performed immediately after determination of absorbance. The excitation wavelength was 785 nm, and emission was measured over the range 790–1,000 nm. Spectra were corrected for the emission spectrum of a blank serum or TCB sample and for the intensity fluctuations of the excitation source. For the samples in TCB, the absolute quantum yield was determined using ICG in ethanol (quantum yield of 13.2%) as the fluorescence standard [23]. For the samples in serum, the absolute quantum yields could not be determined due to the turbidity of the solutions. Therefore, for these samples relative quantum yields were reported comparing nanocolloidal albumin-IRDye 800CW to ICG.

### Preparation of injectable ICG/HSA solution

The noncovalent ICG/HSA injectate was mixed as described by Ohnishi et al. [16]. To 33.5 mg HSA dissolved in 50 ml phosphate-buffered saline (pH 7.4) was added 0.16 ml of a 2.5-mg/ml ICG solution to achieve a final concentration of 10 μM each (1:1 molar ratio). After gentle mixing the ICG/HSA solution was ready for in vivo use.

### In vivo experiments

**Fig. 2:**
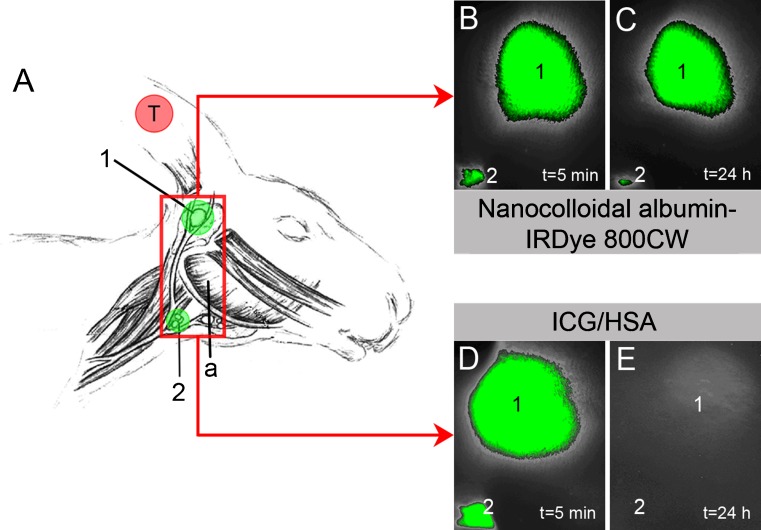
In vivo evaluation of nanocolloidal albumin-IRDye 800CW. **a** Rabbit VX2 auricular carcinoma model. The *red box* represents the field of view of images **b–e**. **b**, **c** NIR fluorescence image of fluorescence-labelled lymph nodes obtained 5 min (**b**) and 24 h (**c**) after peritumoral injection of nanocolloidal albumin-IRDye 800CW (exposure time 500 ms). **d**, **e** NIR fluorescence image of fluorescence-labelled lymph nodes obtained 5 min (**d**) and 24 h (**e**) after peritumoral injection of ICG/HSA (exposure time 1000 ms) (*1* parotid lymph node, *2* caudal mandibular lymph nodes, *a* angle of mandible, *T* tumour)

For in vivo evaluation, New Zealand white rabbits bearing auricular VX2 carcinomas were used. This animal model (Fig. 2a) is attractive for evaluating SN detection procedures in HNSCC, since after inoculation of tumour cells into the external ear the developing tumours tend to metastasize lymphatically. The first lymph node that is metastatically involved in this model is the parotid lymph node, the “SN”; caudal mandibular lymph nodes are considered to be higher tier lymph nodes in this model. The consistent lymphogenic metastatic spread of the tumour and the accessibility of the lymph nodes for surgery make this a very suitable model for evaluation of SN detection [18, 19]

Auricular VX2 carcinoma cell suspensions (0.15–0.25 ml containing 25–30 × 10^6^ VX2 tumour cells), obtained after intramuscular passaging of tumours via the hind limb in other rabbits, were injected into both ears essentially as described by Dünne et al. [19]. Experiments were performed 1–2 weeks after injection of the VX2 tumour cell suspension. Before injection of the tumour cell suspension and of the fluorescent tracer as well as during imaging procedures, animals were anaesthetized with a combination of dexmedetomidine (0.5 mg/kg, Dexdomitor) and ketamine (100 mg/ml, Ketamine 10%). Animals were killed with an overdose of pentobarbital (200 mg/ml, Euthasol 20%) administered intravenously under anaesthesia. All animal experiments were performed in accordance with Dutch animal welfare regulations and Dutch national law (*Wet op de dierproeven*, Stb 1985, 336).

The fluorescent tracers nanocolloidal albumin-IRDye 800CW (six tumours) and ICG/HSA (six tumours) were always administered via four peritumoral subcutaneous injections of 0.25–0.5 ml each and the total injected volume was at least 1 ml. Early NIR fluorescence imaging was performed during injection and was continued until 30 min after injection; late NIR fluorescence image registration was performed after 24 h, with the same imaging settings and positioning of the animal, to allow evaluation of long-term retention of the tracer in the draining lymph nodes. After the late imaging experiments, the animals were killed, and NIR fluorescence-guided resection of fluorescent lymph nodes was performed.

### NIR fluorescence imaging

NIR fluorescence imaging was performed using a Fluobeam NIR imaging system (Fluoptics, Grenoble, France), which is suitable for preclinical as well as clinical applications. This system is compact and portable. It consists of two parts: a control unit with a laser source emitting at 785 nm and a power supply for light-emitting diodes (LEDs), next to an optical head with a highly sensitive charge-coupled device camera and white LEDs for field illumination. The laser beam is fibre-guided from the control unit to the optical head. The laser beam was spread to reach a 6-cm spot diameter at the working distance of 17 cm. The power density of the laser irradiation was 96 μW/mm^2^. The NIR fluorescence image was 696 × 512 pixels and provided a resolution of two line pairs per millimetre allowing visualization of submillimetre structures. The imaging settings of the Fluobeam were adjusted to obtain the best view on the monitor and the best fluorescence-to-background ratio.

## Results

### Preparation and characterization of nanocolloidal albumin-IRDye 800CW

**Fig. 3:**
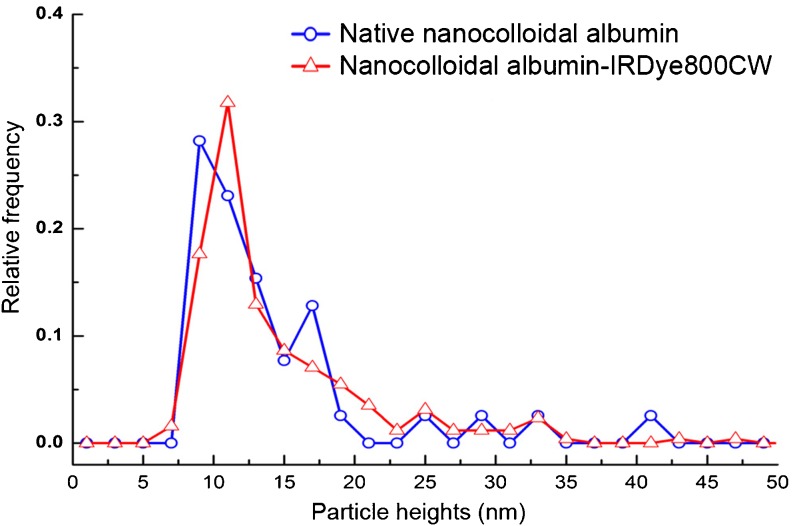
Size distribution of nanocolloidal albumin-IRDye 800CW and native nanocolloidal albumin as assessed by AFM imaging. AFM measured the height of a particle, which represents particle size

Coupling of IRDye 800CW to nanocolloidal albumin as well as native HSA resulted in a conjugation yield of about 50% as determined by a fluorescence spectrophotometer (precipitation method) and/or HPLC. As a result, on average 1.4 IRDye 800CW molecules were coupled per HSA molecule (about 67 kDa) in the nanocolloidal albumin particles. Buckle et al. [24] estimated the molecular weight of a nanocolloidal albumin particle to be about 670 kDa, and this means that a nanocolloidal albumin particle contained on average 14 covalently bound dye molecules. The presence of Tween-80 in the reaction mixture as well as in the PD10 column eluent minimized the amount of associated noncovalently bound dye in the end product, as was shown by control reactions with hydrolysed IRDye 800CW and nanocolloidal albumin (<3%). In general, nanocolloidal albumin-IRDye 800CW appeared to contain <2% of free IRDye 800CW after purification, and this percentage did not change during 120 h of storage. The particle size distributions are shown in Fig. 3. The average size of the nanocolloidal albumin-IRDye 800CW was similar to the average size of native nanocolloidal albumin: 14.6 ± 0.4 nm and 14.1 ± 1.5 nm, respectively. In serum, the relative quantum yields of nanocolloidal albumin-IRDye 800CW and ICG were 0.96 and 1.0, respectively. Absolute quantum yields of nanocolloidal albumin-IRDye 800CW and ICG in TCB were 8.0 ± 0.2% and 8.6 ± 0.1%, respectively.

### In vivo experiments

**Fig. 4:**
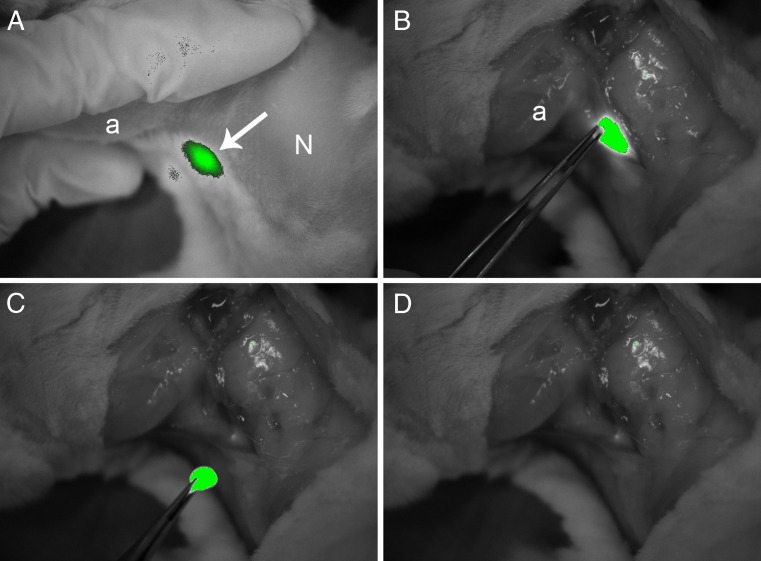
Noninvasive real-time NIR fluorescence detection of a fluorescence-labelled lymph node in the neck of a rabbit. **a** Preoperative image after administration of nanocolloidal albumin-IRDye800CW (*a* angle of mandible, *arrow* lymph node, *N* neck). **b** Intraoperative image after preparation of a skin flap (*a* angle of mandible). **c**, **d** Removal of the lymph node (**c**) after which no fluorescence signal is detectable (**d**) indicating complete removal of fluorescence-containing tissues. Note the high contrast between the fluorescent lymph node and the surrounding tissue. Exposure time in all images was 200 ms

In all experiments draining lymph nodes (parotid lymph node and caudal mandibular lymph node) became visible within 5 min by noninvasive imaging of either nanocolloidal albumin-IRDye 800CW (Fig. 2b) or ICG/HSA (Fig. 2d). After 24 h, noninvasive NIR fluorescence imaging was still able to identify the same 12 draining lymph nodes (parotid and caudal mandibular lymph nodes) in six of six tumours (100%) injected with nanocolloidal albumin-IRDye 800CW, without a decrease in signal intensity and without an increase in background fluorescence (Fig. 2c). However, after 24 h, SNs identified with ICG/HSA during early imaging showed an observable decrease in fluorescence signal, while in 4 of 12 lymph nodes (33%) the fluorescence signal was completely absent (Fig. 2e). Even after open surgery with adjustment of the Fluobeam camera settings (e.g. highest exposure time), these lymph nodes were not detectable. Once a fluorescence signal was detected, it was easy to excise the SN because of the clear delineation of the fluorescence-labelled lymph nodes and the high target-to-background ratio (Fig. 4).

## Discussion

We describe here the covalent conjugation of the fluorescent dye IRDye 800CW to nanocolloidal albumin. The method is very simple and reproducible, and leads to about 14 IRDye 800CW groups per nanocolloidal albumin particle, yielding a strong fluorescence signal intensity. In addition, the size of the nanocolloidal albumin particles was not altered after conjugation to IRDye 800CW, and was shown to be comparable with that of the gold standard ^99m^Tc-Nanocoll [25]. In vivo experiments in a lymphogenic metastatic animal model showed noninvasive NIR fluorescence detection of the SN up to 24 h after injection of the tracer, without any loss of fluorescence intensity, while comparative experiments with ICG/HSA showed a strong decrease or complete loss of fluorescence intensity by 24 h after injection. These characteristics of imaging with nanocolloidal albumin-IRDye 800CW offer an attractive alternative to current procedures for SN detection. The feasibility of NIR fluorescence-guided SN detection has been demonstrated in a variety of tumour types where ICG was used as the fluorescent tracer [6–13, 26, 27]. To date, however, there are just a few small studies which aimed to compare ICG-based SN detection with the standard of care ^99m^Tc-Nanocoll and patent blue. Larger more adequately powered clinical trials to address whether NIR fluorescence imaging alone can replace radiocolloids and/or blue dyes are ongoing [9].

A critical aspect in the use of NIR fluorescence imaging is its limited tissue penetration, which might hamper SN detection in the neck when a thick layer (>1 cm) of overlaying tissue is present as might be found in obese patients. This aspect is very important when considering the use of fluorescence imaging as the sole technique for SN detection. A particular drawback of the use of ICG is its small particle size, which reduces its retention time in the SN and results in rapid spread to higher tier nodes. If a tracer migrates to higher tier nodes, the practical inconvenience arises that nonsentinel lymph nodes are incorrectly identified as SN, leading to the risk of missing a metastatic (real) SN and the resection of more nodes than necessary. This is particularly challenging in the complex anatomy of the neck. In preclinical studies it was demonstrated that ICG adsorption to HSA improves its performance as a lymphatic tracer. However, a clinical randomized, double-blind trial comparing ICG with or without albumin premixing for SN detection in breast cancer patients did not reveal any benefit of ICG premixed with HSA [28].

Buckle et al. developed a so-called self-assembled multimodal complex, which consists of ICG noncovalently associated with ^99m^Tc-Nanocoll. Similar migration properties of ICG and ^99m^Tc-Nanocoll were observed immediately after injection [24]. However, most probably because of the noncovalent interaction of ICG and ^99m^Tc-Nanocoll, dissociation of ICG from ^99m^Tc-Nanocoll was observed, resulting in fluorescent lymph nodes that did not contain radioactivity and therefore could not be considered SNs [29]. Although the clinical feasibility of SN detection with this multimodal complex has been demonstrated [30], the approach as a whole still suffers from the same intrinsic limitations related to ICG as described above.

Our developed technical protocol for labelling and purification ensures that >98% of the IRDye 800CW is covalently bound to the nanocolloidal albumin. In this study, in order to mimic the clinically most relevant situation, we determined the quantum yield of nanocolloidal albumin-IRDye 800CW relative to that of ICG in human serum with a composition resembling lymphatic fluid, i.e. serum diluted with TCB, and demonstrated that the quantum yields of nanocolloidal albumin-IRDye 800CW and ICG are very similar. The absolute quantum yields of nanocolloidal albumin-IRDye 800CW and ICG in TCB were also similar (8.0% vs. 8.6%, respectively). Therefore, our results do not support the reported limitation in the utility of nanocolloidal albumin-IRDye 800CW as a consequence of intra- and intermolecular quenching [16].

Our in vivo experiments exhibited strong fluorescence signals (Fig. 4). Moreover, nanocolloidal albumin-IRDye 800CW showed superior SN retention in our lymphogenic metastatic animal model, and demonstrated comparable particle size and similar kinetics to those we recently described for the SPECT tracer ^99m^Tc-Nanocoll and the novel PET tracer ^89^Zr-nanocolloidal albumin in the same animal model [25]. With the new IRDye 800CW tracer, parotid and caudal mandibular lymph nodes became visible within 5 min of injection, remained clearly visible until at least 24 h after injection without an increase in local background activity, and the information provided with this conjugate was fully congruent with that obtained using the PET tracer ^89^Zr-nanocolloidal albumin at 24 h (see supplementary Fig. S2). This makes possible the direct clinical comparison of the detection of SN using nanocolloidal albumin-IRDye 800CW with the standard of care ^99m^Tc-Nanocoll and patent blue in a 2-day procedure. In such a first-in-human clinical trial, nanocolloidal albumin-IRDye 800CW can simply be coinjected with ^99m^Tc-Nanocoll 24 h before SN removal, since the logistic advantage of nanocolloidal albumin-IRDye 800CW is its potential as an off-the-shelf product. The first clinical objective is the assessment of the SN detection rate and sensitivity of fluorescence imaging, because the dose of nanocolloidal album-IRDye 800CW might be critical as was previously observed for ICG. Nevertheless, we think that the excellent SN fluorescence imaging results obtained in the lymphogenic metastatic animal model can be translated to the human situation in which the same amount of injected volume (1–2 ml) can be used. Although lymph nodes in the head and neck region are often located superficially, the major challenge is expected to be the detection of deep-seated SNs. These trials will determine whether fluorescence detection with nanocolloidal albumin-IRDye 800CW can either replace just the intraoperative blue dye or the gamma probe procedure. Ideally, it has to be shown whether it can also replace preoperative scintigraphic imaging completely resulting in a one-step procedure of NIR fluorescence imaging that takes place entirely during surgery.

## Conclusion

Nanocolloidal albumin-IRDye 800CW is a promising next-generation fluorescent tracer with characteristics that would allow intraoperative SN detection even after a longer interval of time. This makes a reliable comparison between the use of fluorescence-guided detection and the conventional radioactive-guided method possible.

## Electronic supplementary material

Below is the link to the electronic supplementary material.

Fig. S1 (DOCX 342 kb)

Fig. S2 (DOCX 201 kb)
