# Supplementary material for: Nanocolloidal albumin-IRDye 800CW: a near-infrared fluorescent tracer with optimal retention in the sentinel lymph node
Source: Eur J Nucl Med Mol Imaging. 2012 Feb 17;39(7):1161–8. doi: 10.1007/s00259-012-2080-5 (PMC3369133; doi:10.1007/s00259-012-2080-5)
Supplement: Supplementary file 1 — (DOCX 342 kb) [file 259_2012_2080_MOESM1_ESM.docx]

ELECTRONIC SUPPLEMENTARY MATERIAL

**Nanocolloidal albumin-IRDye800CW: a near-infrared fluorescent tracer with optimal retention in the sentinel lymph node**

**Fig. S1** (**A**) HPLC chromatogram of IRDye 800CW-NHS ester in PBS, pH=8.5, after 5 min of incubation in which about 20% of the NHS-ester was already been hydrolyzed (peak 1 at Rt=50.4 min). Peak 2 at Rt ≈78 min shows the IRDye 800CW-NHS ester. (**B**) HPLC Chromatogram of IRDye800CW-NHS ester after 2 h incubation at 35 degrees, which demonstrates that >95% of the NHS-ester has been hydrolyzed
