# Supplementary material for: Nanocolloidal albumin-IRDye 800CW: a near-infrared fluorescent tracer with optimal retention in the sentinel lymph node
Source: Eur J Nucl Med Mol Imaging. 2012 Feb 17;39(7):1161–8. doi: 10.1007/s00259-012-2080-5 (PMC3369133; doi:10.1007/s00259-012-2080-5)
Supplement: Supplementary file 2 — (DOCX 201 kb) [file 259_2012_2080_MOESM2_ESM.docx]

**Fig. S2** (**A**) Rabbit VX2 auricular carcinoma model. (**B**) PET-CT image obtained 5 min after peritumoural injection of 5 MBq of the PET-tracer ^89^Zr-nanocolloidal albumin. (**C**) PET image of the same animal obtained 24 h after injection of ^89^Zr-nanocolloidal albumin demonstrating clear retention of this tracer at that moment with visualisation of a connecting lymphatic vessel (arrow). 1 = parotid lymph node; 2 = caudal mandibular lymph nodes; a = angle of mandible; T = tumour
